# *S*-Ethyl-*N,N*-dipropylthiocarbamate Exposure and Cancer Incidence among Male Pesticide Applicators in the Agricultural Health Study: A Prospective Cohort

**DOI:** 10.1289/ehp.11371

**Published:** 2008-06-26

**Authors:** Dana M. van Bemmel, Kala Visvanathan, Laura E. Beane Freeman, Joseph Coble, Jane A. Hoppin, Michael C.R. Alavanja

**Affiliations:** 1 Division of Cancer Epidemiology and Genetics, National Cancer Institute, National Institutes of Health, Department of Health and Human Services, Rockville, Maryland, USA; 2 Department of Epidemiology and Oncology, The Johns Hopkins Medical Institutions, Baltimore, Maryland, USA; 3 Epidemiology Branch, National Institute for Environmental Health Sciences, National Institutes of Health, Department of Health and Human Services, Research Triangle Park, North Carolina, USA

**Keywords:** agriculture, cancer, EPTC, herbicide, neoplasms, occupational exposure, pesticides, thiocarbamates

## Abstract

**Background:**

The Agricultural Health Study (AHS) is a prospective cohort study of licensed pesticide applicators from Iowa and North Carolina enrolled between 1993 and 1997. EPTC (*S*-ethyl-*N,N*-dipropylthiocarbamate) is a thiocarbamate herbicide used in every region of the United States. The U.S. Environmental Protection Agency reports that EPTC is most likely not a human carcinogen; however, the previous epidemiologic data on EPTC exposure and cancer risk were limited.

**Objectives:**

The purpose of this study was to examine cancer incidence and EPTC use in 48,378 male pesticide applicators enrolled in the AHS.

**Methods:**

We estimated the rate ratio (RR) and 95% confidence intervals (CIs) for all cancers and selected cancer sites using Poisson regression. We assessed EPTC exposure using two quantitative metrics: lifetime exposure days and intensity-weighted lifetime exposure days, a measure that accounts for application factors that modify personal exposure likelihood.

**Results:**

Among the 9,878 applicators exposed to EPTC, 470 incident cancer cases were diagnosed during the follow-up period ending December 2004 compared with the 1,824 cases among individuals reporting no use. Although EPTC was associated with colon cancer in the highest tertile of both lifetime exposure days and intensity-weighted lifetime days (RR = 2.09; 95% CI, 1.26–3.47 and RR = 2.05; 95% CI, 1.34–3.14, respectively) and the trend test was < 0.01 for both, the pattern of RR was not monotonic with increasing use. There was a suggestion of an association with leukemia. No other associations were observed.

**Conclusion:**

In this analysis, EPTC use appeared to be associated with colon cancer and leukemia. However, given the relatively small number of cases in the highest exposure tertile, results should be interpreted with caution, and further investigations are needed.

EPTC, or *S*-ethyl-*N-N*-dipropylthiocarbamate, is a preemergence and early postemergence thiocarbamate herbicide used to manage the growth of annual weeds including broadleaves, grasses, and sedges. EPTC is widely applied with the safener dichlormid (2,2-dichloro-*N,N*-di-2-propenylacetamide) (Abu-Qare and Duncan 2002) to reduce crop-specific phytotoxicity. EPTC is used in every region of the United States in the agricultural production of a wide variety of food crops including corn, potatoes, dry beans, and alfalfa. In 1999, EPTC was the 19th most commonly used pesticide active ingredient in U.S. agriculture [U.S. Environmental Protection Agency (EPA) 2002]. The U.S. EPA (2006) estimates that the total amount of EPTC used has decreased from an average of 21 million pounds in 1987 to 9 million pounds in 1999. States reporting the highest annual use of EPTC between 1995 and 1997 were in the northern Great Plains; Iowa, Nebraska, Minnesota, and South Dakota (U.S. Geological Survey 2004). EPTC is available as an emusifiable concentrate and in a granular formulation. Depending on the formulation, EPTC can be applied by disking, soil injection, and spray. EPTC has a relatively short half-life in soil ranging from 2 to 18 days, depending on microbial conditions. Degradation of EPTC can occur through chemical, biologic, and photo-chemical processes (Abu-Qare and Duncan 2002; Nagy et al. 1995).

Because of its volatile nature and short soil half-life, EPTC is not persistent and does not bioaccumulate up the food chain. Because EPTC does not remain in the environment, the primary route for human exposure is likely nondietary, through dermal contact and inhalation (U.S. EPA 1999). The U.S. EPA has classified EPTC as a general use pesticide that is moderately toxic (Toxicity Category III) via oral and dermal routes (U.S. EPA 1999). In subchronic and chronic studies performed in both rats and dogs, EPTC exposure resulted in cardiomyopathy and neurotoxicity in the central and peripheral nervous systems (U.S. EPA 2006). Toxicity occurs through the byproducts of EPTC metabolism, which inhibit the enzymatic activities of aldehyde dehydrogenase and cholinesterase (Smulders et al. 2003, 2004; Staub et al. 1995; Zimmerman et al. 2004).

Using the 1999 *Guidelines for Carcinogen Risk Assessment* and the Ames mutagenicity test (U.S. EPA 1999), the U.S. EPA reports that EPTC is most likely not a carcinogen. This review was based on long-term studies in small rodents and short-term mutagenicity studies (Dickie 1987; Tisdel 1986). Similar work using human peripheral lymphocytes also indicated that EPTC and other thiocarbamates, when applied directly to cells, do not induce DNA damage (Calderon-Segura et al. 1999, 2007). However, EPTC exerts its herbicidal effects after metabolic transformation into its sulfoxide and sulfone derivatives produced by oxidation reactions (Hubbell and Casida 1977). Recent animal studies found that EPTC sulfoxide can form DNA adducts in rat hepatocytes (Zimmerman et al. 2004) and induces DNA damage in human lymphocytes (Calderon-Segura et al. 2007). In mammals, the same reactive electrophilic intermediates are formed through sulfoxidation and oxidation reactions as part of the normal detoxification pathways (Chen and Casida 1978; Hubbell and Casida 1977). How efficiently humans metabolize and excrete EPTC and its metabolites remains unclear.

The epidemiologic data on EPTC exposure and cancer risk are limited. An earlier study using data unrelated to the Agricultural Health Study (AHS) reported a modest increased risk of non-Hodgkin lymphoma among farmers who applied carbamate pesticides when compared with nonfarmers (Zheng et al. 2001). Earlier analyses within the AHS report no association between ever using EPTC and prostate (Alavanja et al. 2003), colon, or rectal cancer (Lee et al. 2007). Here, we investigate potential associations between a number of cancer sites and self-reported use of EPTC among pesticide applicators enrolled in the AHS.

## Methods

### Cohort enrollment and follow-up

The AHS is a prospective cohort study of 57,311 licensed pesticide applicators and their spouses (*n* = 32,347) in Iowa and North Carolina (Alavanja et al. 1996). Only private applicators (primarily farmers) were recruited from North Carolina; in Iowa, both commercial and private applicators were enrolled. Commercial applicators include individuals employed by companies that regularly use pesticides. All applicators were recruited between December 1993 and December 1997 from certification sessions that are required to use U.S. EPA–designated “Restricted Use Pesticides.” Participants completed an enrollment questionnaire while attending the certification session. Incident cancer cases were identified by matching cohort information to cancer registry files in both Iowa and North Carolina through December 2004 (AHS data release version P1REL0612; unpublished data). Annually, members of the cohort were matched to the National Death Index (National Center for Health Statistics, Hyattsville, MD) to determine vital status. Current address records were compared with motor vehicle registration records, pesticides license registries from each state agricultural department, and address records from the Internal Revenue Service to verify that participants continued to reside in Iowa or North Carolina. Individuals were censored at the time of death or relocation out of the participating states. Informed consent was obtained from all participants, and the protocol was approved by all appropriate institutional review boards. We excluded female applicators (*n* = 1,563) for this analysis because only 82 reported ever using EPTC, among whom only two cases of cancer were observed.

### Exposure assessment

Exposure to EPTC and other items was assessed through self-report. Questionnaires are available online at http://www.aghealth.org (National Institutes of Health 2004). Detailed information on the use of 22 specific pesticides, including EPTC, was collected in the enrollment questionnaire, and on ever/never use for an additional 28 specific pesticides. In addition, information was obtained on pesticide mixing and application methods, types of equipment repair routinely performed, and use of personal protective equipment. Participants also provided information on lifestyle and other factors associated with an increased risk of developing specific cancer, including smoking history, alcohol use, a first-degree relative diagnosed with cancer, diet, and basic demographic characteristics.

We evaluated EPTC exposure using two metrics: lifetime exposure days and intensity-weighted lifetime exposure days. Lifetime exposure days were calculated as the product of the years an individual mixed or applied EPTC and the number of days in an average year that EPTC was used. The intensity-weighted score was calculated using a published algorithm that accounts for many factors that may influence pesticide exposure (Dosemeci et al. 2002), including the effect of modifying factors such as how often an applicator personally mixed or prepared herbicides, the type of application method used, whether an applicator personally repaired pesticide application equipment, and the type of personal protective equipment used. The precise algorithm is as follows: intensity level = [(mixing status + application method + equipment repair status) × personal protective equipment use]. The intensity score was multiplied by the EPTC lifetime exposure days to obtain a final EPTC intensity-weighted exposure days value.

### Data analysis

Only male pesticide applicators who completed the enrollment questionnaire were included in this analysis. Those with prevalent cancer were excluded (*n* = 1,062), as were pesticide applicators who did not provide information on age (*n* = 1) or EPTC use (*n* = 6,307), restricting the initial analysis to 48,378 licensed pesticide applicators. An additional 50 participants were excluded from the analysis using the intensity-weighted lifetime exposure days because of incomplete data collection on factors related to the algorithm. A sensitivity analysis including these exclusions in the referent group was performed using both exposure metrics. And Poisson regression analysis was performed using the STATA statistical software program (STATA version 9.0; StataCorp, College Station, TX). Based on previous pesticide studies, the first 10 factors listed in Table 1 were put into the all-cancer regression model individually to determine significance (*p* = < 0.05). We then used forward selection to add significant variables to the regression model. Three of the 10 variables evaluated were rejected based on the above criteria. All Poisson regression models were adjusted for age as a categorical variable, race, smoking status, alcohol consumption, state of residence, family history of cancer (not including nonmalignant skin cancer), applicator type (commercial/private), and lifetime days of all pesticide use. Lifetime days of all pesticide use was analyzed as both a categorical and continuous variable. Because there were no significant differences between the two variables, we chose to analyze lifetime days of all pesticide use as a continuous variable. Additional modeling was done to adjust for body mass index as a categorical variable for all cancer and colon cancer.

In cancer-specific analyses, we considered 27 different cancer sites [ICD-O2; *International Classification of Diseases for Oncology, Second Revision* (World Health Organization 1990)]. Except for leukemia and rectal cancer, only cancer sites with at least 20 cases among individuals exposed to EPTC are listed in this report. Those specific sites include combined category of blood (including leukemia, non-Hodgkin lymphoma, Hodgkin lymphoma, multiple myeloma, and other cancers of the blood with no known origin), non-Hodgkin lymphoma, as well as bladder, colon, lung, and prostate cancers. Leukemia (*n* = 18 among those reporting EPTC use) was included based on reports suggesting a potential association between herbicide use and an increase in leukemia risk and chromosomal damage (Georgian et al. 1983; Lee et al. 2004). Rectal cancer (*n* = 14 among those reporting EPTC use) was analyzed based on its anatomic proximity to the colon.

Lifetime exposure days and intensity-weighted lifetime exposure days were categorized into tertiles based on the distribution of exposure among all cancer cases. Both exposure metrics were analyzed using two different referent groups: no EPTC use and the lowest tertile of EPTC use. It has been postulated that pesticide users in the highest exposure tertile may be more similar to applicators in the lowest exposure group than they are to never users (Rusiecki et al. 2004). Therefore, we used the low-exposure group as a second reference group to address potential unknown confounding between the groups. We performed a test for linear trend for both exposure metrics by using the median for each exposure category as the quantitative score. All statistical tests were two sided.

## Results

Selected characteristics of the study population are displayed in Table 1 according to lifetime exposure days to EPTC. The low-exposure group refers to participants in the lowest tertile (< 10 lifetime exposure days), and the high-exposure group is a combination of the top two tertiles (≥ 10 lifetime exposure days). Applicators were primarily white, with > 40% attaining at least a high school diploma. Regardless of exposure, approximately 50% of participants were never smokers, and most reported some alcohol use over the preceding 12 months. In general, the three exposure groups (no exposure, low, and high) were similar with respect to most demographic characteristics, except for the predominance of EPTC use by farmers in Iowa.

EPTC exposure was initially divided into no exposure (never users) and exposed (ever users). For all cancer sites combined, 470 cancer diagnoses were made through December 2004 among the 9,878 applicators exposed to EPTC. In contrast, 1,824 cancers were diagnosed within that same time among those with no exposure (*n* = 38,500). The mean (± SD) age of cancer incidence in the study population was 58 ± 10.3 years with a range of 23–87 years. A small increase in risk for all cancers was observed [rate ratio (RR) 1.14; 95% confidence interval (CI), 1.02–1.27]. No specific cancer was observed to be statistically associated with individuals ever exposed to EPTC versus those never exposed, but the risks for both colon cancer (RR = 1.35; 95% CI, 0.93–1.97) and leukemia (RR = 1.31; 95% CI, 0.75–2.28) were elevated. The risk for rectal cancer was not significant (RR = 0.80; 95% CI, 0.44–1.42).

Table 2 summarizes our analysis investigating the association between cancer incidence and EPTC lifetime exposure days (left panel). We observed an increase in risk when comparing the highest level of exposure for all cancer (RR = 1.28; 95% CI, 1.09–1.50), colon cancer (RR = 2.09; 95% CI, 1.26–3.47), and leukemia (RR = 2.36; 95% CI, 1.16–4.84) with subjects with no exposure. We also observed a significant increasing linear trend for the incidence of these cancers (*p*-trend for all cancer < 0.01, *p*-trend for colon cancer < 0.01, *p*-trend for leukemia = 0.02). When the lowest exposure tertile was used as the referent, we observed a slight attenuation of the point estimate for all cancer (RR = 1.13; 95% CI, 0.92–1.39, *p*-trend = 0.05) and an increase for both colon cancer (RR = 2.76; 95% CI, 1.27–6.00, *p*-trend = 0.03) and leukemia (RR = 2.91; 95% CI, 0.97–8.72, *p*-trend = 0.04).

We also analyzed EPTC exposure by tertiles of intensity-weighted lifetime exposure days. The increased risk associated with EPTC lifetime exposure days and colon cancer remained constant when we used the intensity-weighted lifetime exposure days metric (Table 2, right panel). The associated risks for all cancer and leukemia were slightly attenuated using the intensity-weighted lifetime days exposure scale. When comparing the highest tertile of EPTC exposure with those with no exposure, we found increased risks for all cancer (RR = 1.16; 95% CI, 1.01–1.35), colon cancer (RR = 2.05; 95% CI, 1.34–3.14), and leukemia (RR = 1.87; 95% CI, 0.97–3.59). When the lowest tertile of exposure was used as the referent group, the risk associated with cancer incidence and EPTC intensity-weighted exposure days remained elevated at the highest tertile of EPTC exposure for all cancer (RR = 1.19; 95% CI, 0.95–1.49), colon cancer (RR = 2.59; 95% CI, 1.13–5.97), and leukemia (RR = 3.93; 95% CI, 0.87–17.67).

We also divided the upper tertile of exposure at its median to expand examination of the association between high EPTC exposure and cancer incidence for those cancer sites that had five or more cases within each of the upper two exposure levels. An increase in risk associated with the highest level of EPTC lifetime exposure days (≥ 110) was observed when the no-exposure group was used as the referent for all cancer (RR = 1.30; 95% CI, 1.03–1.63) and colon cancer (RR = 3.55; 95% CI, 1.97–6.42). The linear trend tests for all cancer (*p*-trend = 0.01) and colon cancer (*p*-trend = < 0.01) were also statistically significant. When the low-exposure group was used as the referent, only colon cancer remained significantly elevated (RR = 4.70; 95% CI, 2.03–10.87, *p*-trend = < 0.01). An increase in risk associated with the highest level of EPTC intensity-weighted lifetime exposure days (≥ 333) was observed for colon cancer when the no-exposure (RR = 2.21; 95% CI, 1.27–3.86, *p*-trend = < 0.01) and low-exposure (RR = 2.80; 95% CI, 1.13–6.94, *p*-trend = 0.02) referent groups were used. The risk estimate for all cancer associated with the highest level of EPTC exposure remained elevated using either the no-exposure referent (RR = 1.14; 95% CI, 0.93–1.38, *p*-trend = 0.11) or the low-exposure referent (RR = 1.15; 95% CI, 0.89–1.50, *p*-trend = 0.54). However, the CI for the low-exposure referent includes the null, and the linear trend is no longer significant.

We performed stratified analysis of risk for all cancer by state of residence (Iowa vs. North Carolina). No difference was observed in all cancer risk when comparing the highest tertile of EPTC exposure using either lifetime exposure days or the intensity-weighted lifetime exposure days when stratifying by state of residence. When we included all participants with missing information in the referent group, there was no change in the observed risk estimates for all cancers, colon cancer, and leukemia.

Finally, we modeled cancer risk with pesticides previously reported to be associated with colon cancer risk and those found to be most correlated with EPTC use within the AHS cohort. When the five most highly correlated pesticides were added to the lifetime exposure days model as covariates (butylate, trifluralin, imazethapyr, metribuzin, and dicamba), no significant change was observed in our cancer risk estimates for all cancer, colon cancer, and leukemia. Other work from the AHS reported an increased risk of colon cancer associated with increasing exposure to aldicarb (Lee et al. 2007) and dicamba (Samanic et al. 2006). To determine that our observed risk estimates were not related to these pesticide exposures, we modeled colon cancer risk including both as covariates in our lifetime exposure days model. Similarly, our risk estimate was not markedly changed for colon cancer.

## Discussion

In this study we evaluated lifetime EPTC exposure as a risk factor for developing cancer. We observed a positive association between EPTC exposure and both colon cancer and leukemia. Colon cancer was significantly associated with EPTC exposure for both lifetime exposure days and intensity-weighted exposure days. Leukemia was significantly associated with lifetime exposure days to EPTC and only marginally associated with intensity-weighted lifetime exposure days. Other site-specific analyses (including rectal cancer) were not statistically significant.

Farmers have lower rates of cancer incidence compared with the general population—a phenomenon attributed primarily to lower smoking rates and other lifestyle factors (Alavanja et al. 2005; Blair and Zahm 1991). Despite this, multiple epidemiologic studies have reported links between pesticide use and several different types of cancer, including colon cancer. A case study of farmers in Italy found a marginal association between colon cancer risk and pesticide use among orchard farmers (Forastiere et al. 1993). Cohort studies within the United States have reported an association between colorectal cancer rates and occupational exposure to the herbicide alachlor (Acquavella et al. 1996, 2004), although these observation were based on a relatively small number of cases. Within the AHS cohort, aldicarb (Lee et al. 2007) and dicamba (Samanic et al. 2006) have been associated with an increased risk of colon cancer.

A few studies from the AHS cohort have reported no significant change in colon cancer risk with pesticide use (Alavanja et al. 2003; Beane Freeman et al. 2005; Lee et al. 2007). Recent work by Lee et al. (2007) evaluated the association between ever/never use of 50 different pesticides (including EPTC) and colon cancer. Similar to our study, this report did not find a significant association between ever/never use of EPTC and colon cancer risk [our data: RR = 1.35; 95% CI, 0.93–1.97; Lee et al. (2007) data: odds ratio = 1.2; 95% CI, 0.8–1.8]. Unlike our evaluation, this group did not expand their analysis to quantify an exposure response to EPTC lifetime exposure because it did not fit their criteria for extended analysis. Other studies report a decreased relative risk of colon cancer observed among organochlorine (Purdue et al. 2006) and dichlorophenoxyacetic acid (Lee et al. 2007) applicators in the AHS, although no clear dose–response relationship between increasing pesticide exposure days and colon cancer incidence was observed.

We looked at a number of other factors known to be associated with an increased risk for colon cancer. A family history of colon cancer among first-degree relatives, smoking status, body mass index, and increasing age were all statistically associated with an increased risk of colon cancer among EPTC applicators, but these potential confounders did not alter the observed increase in risk. Similar results were obtained for all cancer and leukemia.

Some epidemiologic studies suggest that leukemia and other immunologically related cancers may be related to pesticide exposure. Organophosphate exposure has been associated with an increased risk of developing non-Hodgkin lymphoma (Cantor et al. 1992; De Roos et al. 2003; Waddell et al. 2001) and leukemia (Beane Freeman et al. 2005; Brown et al. 1990). The carcinogenic effects resulting from organophosphate exposure are hypothesized to be related to altered immune activity by irreversibly inhibiting acetylcholine esterase (Kawashima and Fujii 2003). EPTC is a reversible cholinesterase inhibitor that can also lead to neurotoxicity (Smulders et al. 2003, 2004; U.S. EPA 1999). Cholinesterases catalyze the hydrolysis of the acetylcholine into choline and acetic acid during neurotransmission.

Although the mechanism of inactivation is slightly different, it is plausible that prolonged exposure to thiocarbamates like EPTC may induce a immunogenic effect similar to that observed with the organophosphates by disrupting similar biologic metabolic pathways. Experimental studies are needed to substantiate this hypothesis. Although the AHS is a large cohort with comprehensive exposure assessments, one limitation to this study is the relatively small number of exposed incident cases for certain cancers. These small numbers resulted in unstable risk estimates and limited interpretability of the association between cancer incidence and EPTC exposure. There is also the potential for exposure misclassification because many participants were reporting past and present pesticide exposure. Because the exposure classifications were made before disease diagnosis, any misclassification should be nondifferential and result in an attenuation of observed risk estimates. Although exposure misclassification may exist within our study, evaluation of this issue within the AHS cohort has shown that reporting of pesticide use is similar to other variables described by participants, including diet and alcohol consumption (Blair et al. 2002; Hoppin et al. 2002). Additionally, the exposure scores have been shown to provide a reasonably valid measure of exposure intensity by comparing urine metabolite concentrations with pesticide exposure algorithm results among applicators applying the herbicides 2,4-dichlorophenoxy-acetic acid (2,4-D) (*r* = 0.34, *p* = 0.03) and 4-chloro-2-methylphenoxyacetic acid (MCPA) (*r* = 0.18, *p* = 0.05) (Coble et al. 2005). Another evaluation of the algorithm scores found similar correlations with urinary metabolites for liquid applications of the herbicides glyphosate (*r* = 0.23, 95% CI, −0.07, 0.48), 2,4-D (*r* = 0.25, 95% CI, 0.10–0.54), and liquid applications of the insecticide chlorpyrifos, (*r* = 0.42, 95% CI, 0.01–0.70), but not for granular applications (*r* = −0.44, 95% CI, −0.83 to 0.29) (Acquavella et al. 2006). However, the geometric mean of 3,5,6-trichloro-2-pyridinol (TCPy) (the primary metabolite of chlorpyrifos) concentration in the urine was much lower after granular (10 ppb) compared with liquid (24 ppb) applications of chlorpyrifos. The AHS enrollment questionnaire did not collect information on the frequency with which EPTC was applied as a granular versus liquid formulation. However, among the 46% of EPTC applicators who completed a more detailed take-home questionnaire, 92% reported using a broadcast method to apply herbicides, whereas 37% reported using an in-furrow method. Granular formulations are usually applied using an in-furrow method, whereas liquid application methods are more often applied using a broadcast method. Another limitation to note is that EPTC was first licensed for use in the United States in 1958 (U.S. EPA 1999) and has since been manufactured by a number of companies; therefore, its exact composition as an applied pesticide product may have changed over time and varied among pesticide manufactures. Exposure metrics that account for only duration and intensity do not address possible temporal variability in EPTC product composition. Pesticide applicators are exposed to a number of different chemicals in addition to EPTC. We tried to minimize this potential confounding, adjusting for the possible effects by including total lifetime days of all pesticide use in our model. However, it is plausible that some confounding remained, biasing our risk estimates. Finally, without a strong *a priori* hypothesis to focus our analysis on any one cancer site, we chose to look at multiple cancer sites. This is one of the first epidemiologic studies to comprehensively evaluate the potential association between EPTC exposure and cancer. Given the paucity of available data, we believe it is appropriate to evaluate all cancer sites and to report the results for all cancer sites that we analyzed. Further follow-up is clearly necessary, both in this cohort and in other studies to clarify the patterns of association we observed in this study.

The central strength of this study is its prospective design, allowing for definitive assessments of temporality between pesticide exposure and disease incidence. In addition, detailed information on exposure to EPTC (and other pesticides) was available including information on exposure to 22 different pesticides, the number of days applied per year, use of personal protective equipment, and application method. These factors were components of the different exposure metrics used in this study. Cancer incidence was identified through population-based registries, minimizing the potential for ascertainment bias and information bias. Finally, our study allowed us to analyze one specific pesticide while adjusting for lifetime use of other pesticides and lifestyle variables—points that have not been addressed in previous cohort studies of occupationally exposed workers.

The U.S. EPA (1999) currently reports that EPTC is likely not a human carcinogen based on short- and long-term laboratory studies, yet the previous epidemiologic data on EPTC exposure and cancer risk were limited. This is, to our knowledge, the largest epidemiologic examination of EPTC exposure and cancer risk conducted to date. We provide evidence of an association between the highest category of lifetime EPTC exposure days and cancer risk, specifically for leukemia and colon cancer. However, the evidence for colon cancer is more indicative of a stronger association with EPTC exposure than that presented for leukemia. The small number of leukemia cases (*n* = 18, with the median exposure category having only three cases) among individuals exposed to EPTC limited our ability to interpret the association. As more cancers are diagnosed and the cohort ages, analyses that further explore the potential association between EPTC exposure and cancer incidence should be performed to assess the reproducibility of our observations.

## Figures and Tables

**Table 1:** Enrollment characteristics [no. (%)] of men in the AHS enrolled between 1993 and 1997, by exposure category to EPTC.

Characteristic	No exposure (*n* = 38,500)	Low exposure^a^ (*n* = 3,916)	High exposure^b^ (*n* = 5,962)
Age (years)^c^			
< 40	12,790 (33)	1,249 (32)	2,239 (37)
40–49	10,589 (27)	1,259 (32)	1,951 (32)
50–59	7,891 (20)	826 (21)	1,093 (18)
≥60	7,230 (19)	582 (15)	679 (11)
Race^c^			
White	37,432 (97)	3,882 (99)	5,919 (99)
Nonwhite	968 (2.5)	23 (0.6)	36 (0.6)
Missing	100 (0.3)	11 (0.5)	7 (0.5)
State			
Iowa	24,152 (63)	3,577 (91)	5,471 (92)
North Carolina	14,348 (37)	339 (9)	491 (8)
Smoking			
Never	20,098 (52)	2,219 (57)	3,352 (56)
Former	8,627 (22)	949 (24)	1,357 (23)
Current	8,340 (22)	679 (17)	1,133 (19)
Missing	1,435 (4)	69 (2)	120 (2)
Education^c^			
≤High school	21,656 (56)	1,863 (48)	2,950 (49)
> High school	16,046 (42)	1,985 (51)	2,919 (49)
Missing	798 (2)	68 (2)	93 (2)
Alcohol consumption^c^,^d^			
No use in preceding 12 months	12,536 (33)	861 (22)	1,165 (20)
Self-reported use in preceding 12 months	25,357 (66)	3,030 (77)	4,753 (80)
Missing	607 (2)	25 (1)	44 (1)
Family history of cancer^c^,^e^			
No	23,250 (60)	2,243 (57)	3,564 (60)
Yes	12,881 (33)	1,533 (39)	2,166 (36)
Missing	2,369 (6)	140 (4)	232 (4)
Applicator type			
Private	35,557 (92)	3,679 (94)	4,718 (79)
Commercial	2,943 (8)	237 (6)	1,244 (21)
Currently own or work on farm^d^			
Never	3,560 (9)	144 (4)	823 (14)
Ever	34,409 (89)	3,757 (96)	5,111 (86)
Missing	531 (1)	15 (0.4)	28 (0.5)
Field corn production^d^			
No	12,364 (32)	441 (11)	1,226 (21)
Yes	26,136 (68)	3,475 (89)	4,736 (80)
Person-years (total)	356,402	35,878	55,306
Follow-up (years)^f^	9.16 ± 1.91	9.06 ± 1.80	9.22 ± 1.80
Total lifetime days/year of pesticide application^f^	20.60 ± 28.95	17.63 ± 20.31	33.53 ± 34.25

a Low exposure, 1– 10 lifetime days.

b High exposure, ≥10 lifetime days.

c Values do not equal the total because of rounding differences.

d Reported use at enrollment.

e Family history of first-degree relative with any cancer excluding nonmalignant skin cancers.

f Mean ± SD; reported frequency ranged from 0 to 200 days/year of pesticide application. *p* < 0.001.

**Table 2:** Rate ratios for selected cancer sites by lifetime exposure days to EPTC among male pesticide applicators in the AHS followed through December 2004.

Lifetime exposure days				Intensity-weighted lifetime exposure days			
Cancer site	Cases	RR^a^	95% CI	Cancer site	Cases	RR^a^	95% CI
All cancer				All cancer			
No exposure	1,824	1.00	Referent	No exposure	1,824	1.00	Referent
1 < 9	202	1.13	0.98–1.31	1 > 47	118	0.98	0.82–1.19
10 – 49	94	0.96	0.78–1.19	48–111	116	1.27	1.05–1.54
≥50	174	1.28	1.09–1.50	≥112	234	1.16	1.01–1.35
		*p*-Trend* = < 0.01				*p*-Trend* = 0.02	
		*p*-Trend** = 0.05				*p*-Trend** = 0.28	
Bladder				Bladder			
No exposure	85	1.00	Referent	No exposure	85	1.00	Referent
1 < 9	8	1.02	0.48–2.14	1 > 47	3	0.57	0.18–1.83
10 – 49	5	1.16	0.47–2.90	48–111	7	1.71	0.78–3.74
≥50	9	1.21	0.58–2.52	≥112	12	1.18	0.63–2.23
		*p*-Trend* = 0.59				*p*-Trend* = 0.52	
		*p*-Trend** = 0.84				*p*-Trend** = 0.74	
Blood				Blood			
No exposure	182	1.00	Referent	No exposure	182	1.00	Referent
1 < 9	21	1.12	0.71–1.77	1 > 47	14	1.09	0.63–1.90
10 – 49	12	1.14	0.63–2.07	48–111	10	1.05	0.55–1.99
≥50	20	1.46	0.90–2.37	≥112	28	1.37	0.91–2.08
		*p*-Trend* = 0.12				*p*-Trend* = 0.14	
		*p*-Trend** = 0.20				*p*-Trend** = 0.25	
Colon				Colon			
No exposure	140	1.00	Referent	No exposure	140	1.00	Referent
1 < 9	10	0.76	0.40–1.46	1 > 47	7	0.79	0.37–1.70
10 – 49	10	1.44	0.75–2.77	48–111	4	0.61	0.22–1.64
≥50	19	2.09	1.26–3.47	≥112	28	2.05	1.34–3.14
		*p*-Trend* = < 0.01				*p*-Trend* = < 0.01	
		*p*-Trend** = 0.03				*p*-Trend** = 0.01	
Leukemia				Leukemia			
No exposure	59	1.00	Referent	No exposure	59	1.00	Referent
1 < 9	5	0.81	0.32–2.05	1 > 47	2	0.48	0.12–1.96
10 – 49	3	0.89	0.28–2.87	48–111	3	0.97	0.30–3.12
≥50	10	2.36	1.16–4.84	≥112	12	1.87	0.97–3.59
		*p*-Trend* = 0.02				*p*-Trend* = 0.05	
		*p*-Trend** = 0.04				*p*-Trend** = 0.05	
Lung				Lung			
No exposure	181	1.00	Referent	No exposure	244	1.00	Referent
1 < 9	17	1.31	0.79–2.19	1 > 47	10	1.10	0.55–2.17
10 – 49	3	0.44	0.14–1.37	48–111	11	1.46	0.76–2.80
≥50	12	1.02	0.55–1.89	≥112	16	0.78	0.44–1.41
		*p*-Trend* = 0.92				*p*-Trend* = 0.45	
		*p*-Trend** = 0.92				*p*-Trend** = 0.36	
Melanoma				Melanoma			
No exposure	70	1.00	Referent	No exposure	70	1.00	Referent
1 < 9	6	0.81	0.35–1.88	1 > 47	5	0.98	0.39–2.45
10 – 49	11	2.53	1.32–4.87	48–111	5	1.27	0.51–3.18
≥50	5	0.79	0.31–2.02	≥112	12	1.35	0.71–2.55
		*p*-Trend* = 0.89				*p*-Trend* = 0.35	
		*p*-Trend** = 0.81				*p*-Trend** = 0.35	
Non-Hodgkin lymphoma				Non-Hodgkin lymphoma			
No exposure	83	1.00	Referent	No exposure	83	1.00	Referent
1 < 9	10	1.16	0.59–2.26	1 > 47	8	1.36	0.65–2.84
10 – 49	7	1.45	0.66–3.18	48–111	4	0.90	0.33–2.48
≥50	5	0.79	0.31–2.01	≥112	10	1.07	0.55–2.12
		*p*-Trend* = 0.72				*p*-Trend* = 0.89	
		*p*-Trend** = 0.62				*p*-Trend** = 0.97	
Prostate				Prostate			
No exposure	733	1.00	Referent	No exposure	733	1.00	Referent
1 < 9	97	1.31	1.06–1.63	1 > 47	51	1.02	0.77–1.36
10 – 49	36	0.91	0.65–1.27	48–111	60	1.61	1.24–2.11
≥50	61	1.17	0.89–1.53	≥112	82	1.05	0.83–1.33
		*p*-Trend* = 0.31				*p*-Trend* = 0.55	
		*p*-Trend* = 0.62				*p*-Trend** = 0.24	
Rectum				Rectum			
No exposure	73	1.00	Referent	No exposure	73	1.00	Referent
1 < 9	6	0.78	0.34–1.80	1 > 47	4	0.78	0.28–2.15
10–49	2	0.46	0.11–1.90	48–111	2	0.50	0.12–2.07
≥50	6	1.0	0.42–2.40	≥112	8	0.90	0.43–1.92
		*p*-Trend* = 0.91				*p*-Trend* = 0.78	
		*p*-Trend** = 0.36				*p*-Trend** = 0.43	

a Adjusted for age (< 40, 40–49, 50–59, ≥60 years), race, smoking (never, pack-years among former smokers, pack-years among current smokers), alcohol use (ever in the last 12 months), applicator type (commercial or private), family history of cancer, state of residence, and total days pesticide use.

* Trend using the no-exposure group as the referent.

** Trend using the low-exposure group as the referent.
